# Supplementary material for: Psychosocial influences on hypertension in Southeastern Nigeria: A mixed-methods study protocol
Source: PLoS One. 2026 Nov 10;21(11):e0362594. doi: 10.1371/journal.pone.0362594 (PMC13750564; doi:10.1371/journal.pone.0362594)
Supplement: Supplementary file 1 [file pone.0362594.s001.pdf]

S1 File. Participant Characteristics Questionnaire

Study Title: *Psychological Factors Influencing High Blood Pressure in Southeastern Nigeria*

Instructions: Please answer the following questions. Your responses will be kept confidential and used only for research purposes. If you prefer not to answer any question, you may skip it.

Section 1: Personal Information

1. **What is your age?**

   ______ years old

2. **What is your sex?**

   ☐ Male

   ☐ Female

   ☐ Other (please specify): ______________

3. **What is your marital status?**

   ☐ Single

   ☐ Married

   ☐ Divorced/Separated

   ☐ Widowed

   ☐ Other (please specify): ______________

4. **Which of the following best describes your current living arrangement?**

   ☐ Living alone

   ☐ Living with a partner/spouse

   ☐ Living with family members (parents, siblings, etc.)

   ☐ Living with roommates or friends

   ☐ Other (please specify): ______________

5. **What is your highest level of education completed?**

☐ No formal education

☐ Primary school

☐ Secondary school (O-level)

☐ Technical/Vocational education

☐ Undergraduate degree (BSc, HND, etc.)

☐ Postgraduate degree (MSc, PhD, etc.)

☐ Other (please specify): _______________

6. **What is your employment status?**

☐ Employed (Full-time)

☐ Employed (Part-time)

☐ Self-employed

☐ Unemployed

☐ Retired

☐ Student

☐ Other (please specify): _______________

7. **Is your job mostly sedentary or physically active?**

☐ Mostly sedentary (e.g., desk job, driving)

☐ Moderately active (e.g., standing, light movement)

☐ Physically active (e.g., manual labour, walking most of the day)

8. **What is your average monthly income? (in Nigerian Naira - ₦)**

☐ Less than ₦50,000

☐ ₦50,000 – ₦100,000

☐ ₦100,001 – ₦200,000

☐ ₦200,001 – ₦300,000

☐ ₦300,001 – ₦500,000

☐ More than ₦500,000

☐ Prefer not to say

9. **What is your current residential area?**

☐ **Urban** (towns and cities, e.g., busy places with lots of buildings, shops, offices, transport, and people)

☐ **Rural** (countryside e.g., villages, fewer people, fewer large buildings)

☐ **Semi-urban** (areas in between countryside and city)

10. **Do you have access to healthcare services in your area?**

☐ Yes, with no difficulty

☐ Yes, but with some difficulty (e.g., long travel distance, high cost)

☐ No, I do not have easy access to healthcare services

11. **Have you ever participated in a research study on psychological factors and high blood pressure before?**

☐ Yes

☐ No

☐ Unsure

Section 2: Health Information

11. **Have you been diagnosed with high blood pressure by a healthcare professional?**

☐ Yes

☐ No

12. **How long have you had high blood pressure?**

☐ Less than 1 year

☐ 1-5 years

☐ 6-10 years

☐ 10+ years

13. **Do you have a family history of high blood pressure?**

☐ Yes

☐ No

☐ Not sure

14. **Are you currently taking medication for high blood pressure?**

☐ Yes

☐ No

If yes, please specify the type(s) of medication you are currently taking. If you answered 'No' to the previous question, please select the 'No medication' option below. If the medication you take is not listed among the options, please select 'Other' and write the name of the medication.

☐ Diuretics (e.g., hydrochlorothiazide)

☐ ACE inhibitors (e.g., enalapril, lisinopril)

☐ ARBs – Angiotensin II receptor blockers (e.g., losartan, valsartan)

☐ Beta-blockers (e.g., atenolol, metoprolol)

☐ Calcium channel blockers (e.g., amlodipine, diltiazem)

☐ Combination medications (e.g., ACE inhibitor + diuretic)

☐ Herbal or traditional medicine

☐ Other (please specify): _______________

15.Do you have any other health conditions? *(Check all that apply)*

☐ Diabetes

☐ Heart disease

☐ Depression

☐ Anxiety

☐ None

☐ Other (please specify): _______________

Section 3: Lifestyle and Behavioural Factors

16. **How often do you engage in physical activity?**

☐ Rarely/Never

☐ 1-2 times per week

☐ 3-5 times per week

☐ Daily

17. **Do you smoke?**

☐ Never smoked

☐ Former smoker

☐ Current smoker

18. **How often do you consume alcohol?**

☐ Never

☐ Occasionally

☐ Frequently

☐ Daily

19. **How would you describe your diet?**

☐ Mostly unhealthy

☐ Somewhat healthy

☐ Mostly healthy

20. **How would you rate your stress level?**

☐ Low

☐ Moderate

☐ High

21. **Which coping strategy do you use most often?**

☐ Problem-solving (actively working to fix problems)

☐ Emotional coping (seeking emotional support)

☐ Avoidance (ignoring or distancing from stress)

☐ Other (please specify): ________________

22. **Do you have a strong support system (family, friends, community)?**

☐ Yes

☐ No

☐ Not sure

Section 4: Geographic and Cultural Context

23. **Which state do you currently live in?**

☐ Abia

☐ Anambra

☐ Ebonyi

☐ Enugu

☐ Imo

24. **Religious Affiliation (Optional):**

☐ Christianity

☐ Islam

☐ Traditional religion

☐ None

☐ Other: ______________
