# Supplementary material for: Psychosocial influences on hypertension in Southeastern Nigeria: A mixed-methods study protocol
Source: PLoS One. 2026 Nov 10;21(11):e0362594. doi: 10.1371/journal.pone.0362594 (PMC13750564; doi:10.1371/journal.pone.0362594)
Supplement: Supplementary file 2 [file pone.0362594.s002.pdf]

S2 File. Qualitative Interview Questions to Explore Psychological Factors Impacting High Blood Pressure in Southeastern Nigeria

The following interview questions are designed to delve into the psychological factors that may impact individuals living with high blood pressure in Nigeria. These questions aim to uncover personal experiences, perceptions, and coping mechanisms related to stress, anxiety, depression, and other psychological influences. They are structured to allow for an in-depth exploration of both individual and cultural perspectives, and to understand the contextual factors unique to Nigeria. Each question was developed based on established theoretical models and validated psychometric instruments, ensuring alignment with the study's biopsychosocial and stress–coping frameworks as well as cultural appropriateness within the Nigerian context.

1. Health, Literacy and Education

Q: What is your understanding of high blood pressure and its risks? How did you come to know about it? *Prompt: You can describe what you believe high blood pressure is, how it affects health, and where you learned about it (e.g., hospital, family, media).*

Rationale: Assesses health literacy levels, informing the need for targeted educational programs.

Q: What type of information on high blood pressure or mental health would you like to receive that you currently do not have access to? *Prompt: Think about whether you need more information on treatment, stress management, or understanding your diagnosis.*

Rationale: Directly identifies gaps in health knowledge and sources of information, guiding educational content development.

2. General Understanding and Perception of Personal High Blood Pressure Experience

Q: Can you describe, in your own words, what high blood pressure means to you?
Prompt: Share your personal feelings or beliefs about having high blood pressure.

Q: How did you feel when you were first diagnosed with high blood pressure? *Prompt: You may talk about emotions like fear, confusion, or relief.*

Q: What do you believe are the main causes of high blood pressure in your life?
Prompt: You might mention stress, family history, work, or other life factors

Rationale: These questions aim to understand the participant's basic perception of high blood pressure and its causes, setting the stage for exploring deeper psychological factors.

3. Stress and High Blood Pressure

Q: How would you describe your experience of stress in daily life? *Prompt: Think about situations that make you feel overwhelmed or tense.*

Q: What types of situations or events cause you the most stress? *Prompt: This could include things like work, finances, relationships, family responsibilities, health concerns, or anything else that feels challenging or emotionally demanding. If you don't usually feel stressed, are there still moments that leave you feeling mentally drained or unsettled?*

Q: How do you think stress has impacted your blood pressure? *Prompt: You can reflect on whether your blood pressure increases when stressed.*

Q: How do you usually manage or cope with stress? *Prompt: Share any habits, activities, or support you rely on when feeling stressed.*

Rationale: These questions focus on identifying specific stressors in the participant's life and how they perceive these stressors to impact their high blood pressure. Understanding coping mechanisms provides insight into adaptive or maladaptive responses.

4. Anxiety and Low emotional state

Q: Do you ever feel anxious, worried, or uneasy about your health or life in general? Can you describe what that feels like for you?

Prompt: *You might talk about things like restlessness, overthinking, trouble sleeping, or physical signs like a racing heart or stomach discomfort.*

Q: (Follow-up): How often do you feel this way? *Prompt: Would you say it happens rarely, sometimes, or often?*

Q: How do anxiety or feeling low affect your blood pressure or health? *Prompt: You can talk about whether your emotional state makes it harder to manage your health or take care of yourself — for example, remembering to take medication, eating well, or staying active.*

Q: Have you ever sought help for anxiety or feeling low? If yes, what was your experience? *Prompt: This can include visits to a hospital, talking to a friend, or not seeking help.*

Rationale: These questions aim to explore the presence and impact of anxiety and depression on the participant's blood pressure. Understanding their experiences with mental health can reveal the psychological burden of living with hypertension.

5. Cultural Beliefs and Attitudes

Q: What role do you think culture plays in how people understand and manage high blood pressure? *Prompt: You can mention beliefs in your community about the causes or treatment of high blood pressure.*

Q: Are there traditional or cultural practices you or others use to manage stress or hypertension? *Prompt: These could include herbal remedies, prayers, or rituals.*

Q: How do family or community beliefs affect your own approach to managing blood pressure? *Prompt: Think about whether people encourage or discourage medication or hospital visits.*

Rationale: These questions are designed to uncover cultural influences on the perception and management of hypertension, which is crucial for understanding psychological factors in a Nigerian context.

6. Social Support

Q: Who do you turn to for support when stressed or overwhelmed? *Prompt: This could be family, friends, religious leaders, or no one at all.*

Q: How important is support from others in helping you manage your high blood pressure?

Prompt: This could include support from family, friends, religious or community leaders, healthcare providers, or anyone else in your life. Feel free to share how their support affects your health or daily routine.

(If relevant): Would you say that a lack of support has ever made it harder to manage your high blood pressure? *Prompt: If there were times you felt alone or unsupported, how did that affect your health or ability to manage your condition?*

Rationale: These questions explore the role of social support networks in buffering the psychological impact of hypertension and the consequences of lacking such support.

7. Socio-Economic factors

Q: How does your financial situation affect your ability to manage stress and high blood pressure? *Prompt: You might talk about whether you can afford things like medication, healthy food, or transport to the clinic. If it applies, you can also share whether you receive any financial support—such as help from the government, community programs, or family—and how that affects your ability to care for your health.*

Rationale: This explores the direct impact of economic status on health management, highlighting barriers like access to medication and healthy food that might be financially driven.

8. Coping Mechanisms and Strategies

Q: When you feel stressed or anxious, what do you usually do to feel better? *Prompt: Think about actions like praying, talking to someone, or isolating yourself.*

Q: Can you share specific strategies that help you cope with hypertension mentally or emotionally? *Prompt: Techniques like exercise, meditation, or problem-solving.*

Q: Have you ever used any substances to help you cope with stress or difficult emotions? If so, how do you think this has affected your high blood pressure? *Prompt:*

This could include alcohol, tobacco, recreational drugs, or even the use of medications—whether prescribed or not. You can share whether you feel these habits have helped or made things more difficult in managing your health.

Rationale: These questions focus on identifying both adaptive and maladaptive coping mechanisms that participants use to manage their psychological stress and hypertension.

9. Environmental Stressors

Q: How do your surroundings (e.g., noise, housing, access to care) affect your stress?
Prompt: Consider whether where you live makes it easier or harder to stay healthy.

Q: Can you describe any challenges in your environment that affect your blood pressure? *Prompt: Things like pollution, poor roads, or living far from hospitals.*

Q: How does living in a city or village affect your ability to manage high blood pressure?

Prompt: You might think about access to healthcare services, availability of information, support from others, or the general environment where you live.

Q: Are you aware of any differences in healthcare access between rural and urban areas? *Prompt: You can talk about your own experience or things you've heard from others—for example, differences in the availability of clinics, healthcare workers, medications, or health information.*

Rationale: These questions are aimed at understanding the environmental factors that contribute to psychological stress and hypertension, particularly in the Nigerian context.

10. Personal Stories and Narratives

Q: Can you share a specific time when you are aware your emotional state affected your blood pressure? *Prompt: You can talk about a stressful event and how your BP responded.*

Q: How has living with high blood pressure changed your life mentally or emotionally? *Prompt: Think about changes in your mindset, relationships, or self-esteem.*

Q: What advice would you give to someone newly diagnosed with high blood pressure to manage mental health? *Prompt: Offer suggestions based on what has worked for you.*

Rationale: These open-ended questions allow participants to share personal stories and reflections, providing rich, qualitative data that can reveal the nuanced interplay between psychological factors and hypertension.

11. Perceived Barriers to Psychological Health

Q: What are the biggest challenges to staying mentally healthy while managing high blood pressure? *Prompt: These could include stigma, money, or lack of time.*

Q: Have you faced any challenges in accessing mental health care? What were they? *Prompt: Think about cost, distance, fear, or lack of services.*

Q: How could the Nigerian healthcare system better support people's psychological needs? *Prompt: Suggest what hospitals or communities could do to improve mental health services.*

Rationale: These questions aim to identify perceived barriers to maintaining psychological health and accessing mental health care, which can inform recommendations for healthcare improvements.

12. Recommendations for Improvement

Q: What should be done to better support people with high blood pressure in managing stress, anxiety, or depression? *Prompt: More education? support groups? or free services?*

Q: How can doctors or community leaders help reduce the mental burden of high blood pressure? *Prompt: Share ideas on how people in your community could make a difference.*

Rationale: These questions seek the participant's input on potential improvements in care and support systems, offering insights for practical applications of the research findings.

The references below support the formulation of interview questions by providing relevant theoretical, psychometric, and contextual foundations, ensuring that the qualitative inquiry aligns with the study's conceptual framework and maintains cultural and methodological rigour.

References

1. Adeloye, D., et al., *An estimate of the prevalence of hypertension in Nigeria: a systematic review and meta-analysis.* Journal of hypertension, 2015. **33**(2): p. 230–242. ^https://doi.org/10.1097/HJH.0000000000000413
2. Akinlua, J.T., et al., *Current prevalence pattern of hypertension in Nigeria: A systematic review.* PLOS ONE, 2015. **10**(10): p. e0140021. ^https://doi.org/10.1371/journal.pone.0140021
3. Braun, V. and V. Clarke, *Using thematic analysis in psychology.* Qualitative Research in Psychology, 2006. **3**(2): p. 77–101. ^https://doi.org/10.1191/1478088706qp063oa
4. Byiringiro, S., et al., *Health systems interventions for hypertension management in Sub-Saharan Africa: A systematic review.* PLOS Global Public Health, 2023. **3**(6): p. e0001794. ^https://doi.org/10.1371/journal.pgph.0001794

5. Cohen, S., T. Kamarck, and R. Mermelstein, *A global measure of perceived stress.* Journal of Health and Social Behavior, 1983. **24**(4): p. 385–396. ^ https://doi.org/10.2307/2136404

6. Endler, N.S. and J.D.A. Parker, *Multidimensional assessment of coping: A critical evaluation.* Journal of Personality and Social Psychology, 1990. **58**(5): p. 844–854. ^https://doi.org/10.1037/0022-3514.58.5.844

7. Engel, G.L., *The need for a new medical model: A challenge for biomedicine.* Science, 1977. **196**(4286): p. 129–136. ^https://doi.org/10.1126/science.847460

8. Guest, G., A. Bunce, and L. Johnson, *How many interviews are enough? An experiment with data saturation and variability.* Field Methods, 2006. **18**(1): p. 59–82. ^https://doi.org/10.1177/1525822X05279903

9. House, J.S., *Work stress and social support*. 1981: Addison-Wesley.

10. Kallio, H., et al., *Systematic methodological review: Developing a framework for a qualitative semi-structured interview guide.* Journal of Advanced Nursing, 2016. **72**(12): p. 2954–2965. ^https://doi.org/10.1111/jan.13031

11. Lazarus, R.S. and S. Folkman, *Stress, appraisal, and coping*. 1984: Springer.

12. Leventhal, H., D. Meyer, and D. Nerenz, *The common sense representation of illness danger*. Contributions to medical psychology. Vol. 2. 1980, New York, NY: Pergamon Press.

13. National Bureau of Statistics (NBS). *Nigeria Living Standards Survey (NLSS) 2018–19 (Report)*. 2020; Available from: https://microdata.worldbank.org/index.php/catalog/3827/download/49289.

14. National Health Service (NHS). *High blood pressure (hypertension): Treatment*. 2020 2020 [cited 2025 8 November]; Available from: https://www.nhs.uk/conditions/high-blood-pressure-hypertension/treatment/.

15. Odili, A.N., et al., *Prevalence, awareness, treatment and control of hypertension in Nigeria: Data from a nationwide survey 2017.* Global Heart, 2020. **15**(1): p. 47. ^https://doi.org/10.5334/gh.848

16. Patton, M.Q., *Qualitative research & evaluation methods (4th ed.)*. 2015, Thousand Oaks, CA: SAGE Publications.

17. Sarason, I.G., et al., *A brief measure of social support: Practical and psychometric evaluation of the Social Support Questionnaire.* Journal of Social and Personal Relationships, 1987. **4**(4): p. 497–510. ^https://doi.org/10.1177/0265407587044007

18. World Health Organisation. *Guideline for the pharmacological treatment of hypertension in adults*. 2021; Available from: https://www.who.int/publications/i/item/9789240033986.

19. World Health Organisation. *The WHO STEPwise approach to noncommunicable disease risk factor surveillance (STEPS): Surveillance manual.* 2021; Available from: https://www.who.int/docs/default-source/ncds/ncd-surveillance/steps/steps-manual.pdf?

20. Zigmond, A.S. and R.P. Snaith, *The Hospital Anxiety and Depression Scale.* Acta Psychiatrica Scandinavica, 1983. **67**(6): p. 361–370. ^https://doi.org/10.1111/j.1600-0447.1983.tb09716.x
