# Supplementary material for: Psychosocial influences on hypertension in Southeastern Nigeria: A mixed-methods study protocol
Source: PLoS One. 2026 Nov 10;21(11):e0362594. doi: 10.1371/journal.pone.0362594 (PMC13750564; doi:10.1371/journal.pone.0362594)
Supplement: Supplementary file 3 [file pone.0362594.s003.pdf]

Supplementary Table 1. STROBE Statement completed checklist

SECTION/TOPIC	ITEM No.	STROBE Recommendation	OUR PAPER
**Title and abstract**	1	(*a*) Indicate the study's design with a commonly used term in the title or the abstract	Title page; Abstract – Paragraphs 1–2
		(*b*) Provide in the abstract an informative and balanced summary of what was done and what was found	Abstract – Paragraphs 1–3
**Introduction**			
Background/rationale	2	Explain the scientific background and rationale for the investigation being reported	Introduction – Paragraphs 1–9
Objectives	3	State specific objectives, including any prespecified hypotheses	• Study Hypotheses – Paragraph 1<br>• Aims and Objectives – Paragraphs 1–5
**Methods**			
Study design	4	Present key elements of study design early in the paper	Study Design – Paragraph 1; Figure 1 caption
Setting	5	Describe the setting, locations, and relevant dates, including periods of recruitment, exposure, follow-up, and data collection	• Study Setting & Participants – Paragraphs 1–3<br>• Study Design – Paragraph 2
Participants	6	(*a*) Give eligibility criteria and methods of selection of participants	Study Setting & Participants – Paragraphs 1–3
		(*b*) For matched studies, give matching criteria	Not applicable
Variables	7	Clearly define all outcomes, exposures, predictors, potential confounders, and effect modifiers. Give diagnostic criteria, if applicable	• Participant Characteristics & Measures (Personal, Health, Lifestyle, Geographic sections)<br>• Psychological Questionnaires – Paragraphs 1–5.
Data sources/ measurement	8	For each variable of interest, give sources of data and details of methods of assessment (measurement). Describe comparability of assessment methods if there is more than one group	• Data Collection – Paragraph 1<br>• Participant Characteristics & Measures – All subsections<br>• Psychological Questionnaires – Paragraphs 1–5.
Bias	9	Describe any efforts to address potential sources of bias	• Sampling Strategy – Paragraph 1<br>• Pilot Study – Paragraph 1<br>• Recruitment Procedures – Paragraph 1.
Study size	10	Explain how the study size was arrived at	Sample Size and Power Calculation – Paragraph 1
Quantitative variables	11	Explain how quantitative variables were handled in the analyses. If applicable,	• Quantitative Data Analysis – Paragraph 1

		describe which groupings were chosen and why	• Data Management – Paragraph 1
Statistical methods	12	(*a*) Describe all statistical methods, including those used to control for confounding	Quantitative Data Analysis – Paragraph 1
		(*b*) Describe any methods used to examine subgroups and interactions	• Sampling Strategy – Paragraph 1<br>• Mediation and Moderation Analysis – Paragraph 1
		(*c*) Explain how missing data were addressed	Data Management – Paragraph 1
		(*d*) Explain how loss to follow-up was addressed	Not applicable (cross-sectional design)
		(*e*) Describe any sensitivity analyses	Not applicable (not planned in protocol phase)
**Results**			
Participants	13	(a) Report numbers of individuals at each stage of study—e.g. numbers potentially eligible, examined for eligibility, confirmed eligible, included in the study, completing follow-up, and analysed<br>(b) Give reasons for non-participation at each stage<br>(c) Consider use of a flow diagram	Not applicable (protocol; no data collected yet)
Descriptive data	14	(a) Give characteristics of study participants (e.g. demographic, clinical, social) and information on exposures and potential confounders<br>(b) Indicate number of participants with missing data for each variable of interest<br>(c) *Cohort study*—Summarise follow-up time (e.g., average and total amount)	Not applicable (protocol; no data collected yet)
Outcome data	15	Report numbers of outcome events or summary measures over time	Not applicable (protocol; no data collected yet)
Main results	16	*(a)* Give unadjusted estimates and confounder-adjusted estimates and their precision.<br>(*b*) Report category boundaries when continuous variables were categorized<br>(*c*) If relevant, consider translating estimates of relative risk into absolute risk for a meaningful time period	Not applicable (protocol; no data collected yet)
Other analyses	17	Report other analyses done—e.g. analyses of subgroups and interactions, and sensitivity analyses	Not applicable (protocol; no data collected yet)
**Discussion**			

Key results	18	Summarise key results with reference to study objectives	Not applicable — this is a study protocol, not a completed study.<br>Related objectives are described in:<br>• Aims and Objectives – Paragraphs 1–5<br>• Introduction – Paragraph 9
Limitations	19	Discuss limitations of the study, taking into account sources of potential bias or imprecision. Discuss both direction and magnitude of any potential bias	Not applicable — this is a study protocol; no result-based limitations can be discussed yet.<br>Methodological considerations related to potential bias are described in:<br>• Pilot Study – Paragraph 1<br>• Qualitative Rigour – Paragraph 1<br>• Sampling Strategy – Paragraph 1
Interpretation	20	Give a cautious overall interpretation of results considering objectives, limitations, multiplicity of analyses, results from similar studies, and other relevant evidence	Not applicable — interpretation of findings cannot be provided because no data have been collected.<br>Conceptual foundations and theoretical interpretations guiding the planned study are outlined in:<br>• Introduction – Paragraphs 3–9<br>• Theoretical Framework – All subsections
Generalisability	21	Discuss the generalisability (external validity) of the study results	Not applicable — generalisability relates to study results, which are not yet available.<br>However, considerations affecting the potential reach and representativeness of the planned sample are discussed in:<br>• Study Setting & Participants – Paragraphs 1–3<br>• Sampling Strategy – Paragraph 1
**Other information**			
Funding	22	Give the source of funding and the role of the funders	Funding section (title page manuscript metadata)
