# Supplementary material for: Psychosocial influences on hypertension in Southeastern Nigeria: A mixed-methods study protocol
Source: PLoS One. 2026 Nov 10;21(11):e0362594. doi: 10.1371/journal.pone.0362594 (PMC13750564; doi:10.1371/journal.pone.0362594)
Supplement: Supplementary file 5 [file pone.0362594.s005.pdf]

Inclusivity in global research

PLOS' policy on inclusivity in global research aims to improve transparency in the reporting of research performed outside of researchers' own country or community and ensures that PLOS publications reporting global research adhere to high standards for research ethics and authorship. Authors of relevant research articles may be asked to complete the questionnaire below, which outlines ethical, cultural, and scientific considerations specific to inclusivity in global research. This questionnaire may be requested when researchers have travelled to a different country to conduct research, if research uses samples collected in another country, research with Indigenous populations or their lands, or if research is on cultural artefacts. Researchers travelling to another country solely to use laboratory equipment will not normally be required to complete the questionnaire. However, the questionnaire can be requested at the journal's discretion for any submission – if you have been requested to complete this questionnaire by the PLOS journal you submitted to, please do so.

Please complete the questionnaire below and include this as a Supporting Information file with your manuscript. Note that if your paper is accepted for publication, this checklist will be published with your article in the supporting information files. Please ensure that you reference the checklist in the main body of your manuscript. We suggest adding a subsection 'Inclusivity in global research' to your Methods section and adding the following sentence: "Additional information regarding the ethical, cultural, and scientific considerations specific to inclusivity in global research is included in the Supporting Information (SX Checklist)"

The questions have been designed to be applicable to a wide range of study types, and there are subsections for both human subjects research and non-human subjects research. If any of the questions are not relevant to your research please mark them as "N/A" as appropriate.

Ethical considerations, permits and authorship

This section is applicable to all research types.

Provide details as to who granted permissions and/or consent for the study to take place in the Methods section of your manuscript. This should include the names of **all** ethics boards, governmental organizations, community leaders or other bodies that provided approval for the study. If individuals provided approval refer to these people by their role or title but do not list their name(s).

Reported on page number: pp. 24–25

Ethical approval for this study was obtained from the University of Bradford Research Ethics Committee (Reference: E1322) on 7 August 2025. The study adheres to the Declaration of Helsinki and University of Bradford ethical guidelines.

The study does not involve access to hospital records, clinical databases, biological samples, or clinical interventions. Participants are recruited voluntarily through community organisations, healthcare environments (via study advertisement), churches, and digital platforms.

All data are self-reported via a Jisc-hosted survey or interviews. No identifiable patient data are accessed through healthcare institutions. The study poses minimal risk and all participants provide informed consent prior to participation.

If there were any deviations from the study protocol after approval was obtained please provide details of these changes in the Methods section of your manuscript.

Reported on page number: 22–23

No significant deviations from the approved study protocol have occurred. The study has been conducted in accordance with the approved sequential explanatory mixed-methods design.

Minor refinements were made following pilot testing conducted between November and December 2025 — specifically, small adjustments to survey question wording to improve clarity and reading ease — which did not affect the ethical integrity, core methodology, or scope of the study. These refinements were within the scope of the approved protocol.

Did this study involve local collaborators that are residents of the country where the research was conducted or members of the community studied? If you do not have any authors from said communities, please provide an explanation for this below.

Reported on page number:
pp. 1, 10–12, 30

Yes. The lead researcher and first author (AREO) is of Nigerian origin with direct personal, cultural, and professional ties to Southeastern Nigeria. She has an established understanding of the sociocultural, healthcare, and community contexts of the study region, which informed the study design, recruitment strategies, instrument selection, and planned dissemination activities.

Recruitment for both the quantitative and qualitative components is conducted through healthcare facilities, community-based organisations, churches, and digital platforms across the five Southeastern Nigerian states (Abia, Anambra, Ebonyi, Enugu, and Imo), facilitated through existing community partnerships and networks.

All authors meet PLOS authorship criteria. Author contributions are described in the Author Contributions section of the manuscript.

Everyone listed as an author should meet PLOS' criteria for authorship and all individuals who meet these criteria should be included in the author byline, rather than the acknowledgements. For further information please see the journal's Authorship Policy.

Human subjects research (e.g. health research, medical research, cross-cultural psychology)

Did you obtain written informed consent from a representative of the local community or region before the research took place? How did you establish who speaks for the community? Details of written informed consent obtained from study participants should be reported separately in the Methods section of your manuscript.

Reported on page number:
pp. 24–25

This study recruited individual adult participants with confirmed hypertension across multiple states, settlement types, and community structures in Southeastern Nigeria. It did not involve a single identifiable community authority or collective governance structure that required community-level representative consent prior to the study commencing.

Accordingly, informed consent is obtained directly from each individual participant prior to participation, in line with the Declaration of Helsinki. For quantitative survey participants, consent is obtained electronically — participants review a Participant Information Sheet and consent statement embedded within the Jisc-hosted survey before proceeding. For qualitative interview participants, written or electronic informed consent is obtained prior to each interview. Participants are informed of their right to withdraw at any time without consequence.

This individual consent approach is consistent with standard ethical practice for cross-sectional survey and interview-based research in the Nigerian public health context and was approved by the University of Bradford Research Ethics Committee (Reference: E1322).

How did members of the local community provide input on the aims of the research investigation, its methodology, and its anticipated outcome(s)?

Reported on page number:
pp. 14–16, 22–23

Community input was incorporated into the study design in several ways.

First, the lead researcher (AREO) drew on her direct knowledge of Southeastern Nigerian healthcare contexts, community structures, and cultural frameworks to shape the study aims, instrument selection, and sampling strategy. This insider positionality ensured that the research questions and methodology were grounded in the lived realities of the target population.

Second, a pilot study was conducted with 10 hypertensive adults (two from each of the five Southeastern states) prior to main data collection. Pilot participants provided feedback on the clarity, comprehension, cultural relevance, and usability of the survey instruments. This feedback led to minor refinements in wording to improve accessibility and cultural appropriateness.

Third, recruitment pathways were designed to reflect existing community structures — including healthcare facilities, community-based organisations, churches, and digital platforms — which are trusted channels for health communication and engagement in Southeastern Nigeria.

When engaging with the local community, how did you ensure that the informed consent documents and other materials could be understood by local stakeholders?

Reported on page number:
pp. 11, 15–16, 22–23

All study materials — including the Participant Information Sheet, consent statement, and survey questionnaire — were written in plain English, the official language of Nigeria and the standard language for healthcare communication and national surveys.

The Jisc-hosted survey was designed to maximise accessibility through a smartphone-friendly layout, large font sizes, simple navigation, and clear, non-technical language.

Comprehension and usability were assessed during pilot testing through cognitive debriefing with 10 participants (two per state). Feedback informed minor refinements prior to full data collection. The pilot confirmed that participants could complete the survey accurately within 25–30 minutes.

The study acknowledges that English-language eligibility may underrepresent individuals with very low levels of formal education, and this is noted as a limitation.

Will the findings of the research be made available in an understandable format to stakeholders in the community where the study was conducted (e.g. via a presentation, summary report, copies of publications, etc.)? Please provide details of how this will be achieved.

Reported on page number:
p. 29

Yes. The dissemination plan extends beyond academic publication.

Findings will be submitted as part of a doctoral thesis at the University of Bradford and published in peer-reviewed journals and presented at international conferences.

Key findings and recommendations will also be shared with Nigerian health organisations, participating institutions, media, and community members through summary reports, policy briefs, and community meetings. These outputs will be written in accessible, non-technical language suitable for community stakeholders.

Non-human subjects research using specimens/ animals collected as part of the study, or those housed in archival collections. Examples include archaeology, paleontology, botany and zoology.

Did the permission you obtained from a local authority to perform the study include an agreement on access to outputs and benefit sharing? This may include procedures to enable fair distribution of the benefits and resources arising from the research performed. Please include any details of Prior Informed Consent and Benefit Sharing Agreements obtained. These may be required by field-specific regulations, for example the Convention on Biological Diversity (CBD) and the associated Nagoya Protocol.

Reported on page number:
N/A

Not applicable. This study involves human participants only. No specimens, animals, archaeological materials, or cultural artefacts were used.

If the material used in your study was imported, please A) provide the year it was imported and B) indicate whether permits were obtained to import/export the materials used, C) provide details of any permits obtained. If this information is not available, please indicate this.

Reported on page number:
N/A

Not applicable. This study involves human participants only. No specimens, animals, archaeological materials, or cultural artefacts were used.

If you used archival specimens, please state how the material used in your study was acquired by the institute it is held in and provide details of any permits obtained for the original excavations/ sample collection. If this information is not available, please indicate this.

Reported on page number:
N/A

Not applicable. This study involves human participants only. No specimens, animals, archaeological materials, or cultural artefacts were used.

How was the potential cultural significance of the materials collected in your study to local communities considered in your research design? Were Indigenous peoples and/or local researchers and institutions involved with archaeological excavations / collection of specimens? If so, please provide a description of their involvement.

Reported on page number:
N/A

Not applicable. This study involves human participants only. No specimens, animals, archaeological materials, or cultural artefacts were used.

If your manuscript includes photographs of human remains please indicate whether authors obtained permission from descendants or affiliated cultural communities to do so.

Reported on page number:
N/A

Not applicable. This study involves human participants only. No specimens, animals, archaeological materials, or cultural artefacts were used.
